# Decreased Serum Antibodies Against Oxidized Low-Density Lipoprotein Levels Are Associated with Peripheral Arterial Disease in Patients Undergoing Peritoneal Dialysis

**DOI:** 10.3390/medicina62040691

**Published:** 2026-04-03

**Authors:** Chih-Hsien Wang, Liang-Te Chiu, Yu-Hsien Lai, I-Min Su, Bang-Gee Hsu

**Affiliations:** 1 Department of Medicine, School of Medicine, Tzu Chi University, Hualien 97004, Taiwan; 2 Division of Nephrology, Hualien Tzu Chi Hospital, Buddhist Tzu Chi Medical Foundation, Hualien 97004, Taiwan; 3 Division of Nephrology, Department of Internal Medicine, Dalin Tzu Chi Hospital, Buddhist Tzu Chi Medical Foundation, Chiayi 62247, Taiwan; 4 Department of Pharmacology, School of Medicine, Tzu Chi University, Hualien 97004, Taiwan; 5 Department of Anesthesiology, Dalin Tzu Chi Hospital, Buddhist Tzu Chi Medical Foundation, Chiayi 62247, Taiwan; 6 Institute of Medical Sciences, Tzu Chi University, Hualien 97004, Taiwan

**Keywords:** anti-oxidized low-density lipoprotein antibodies, ankle–brachial index, peritoneal dialysis, peripheral artery disease, C-reactive protein

## Abstract

*Background and Objectives*: Peripheral arterial disease (PAD) is highly prevalent in patients with end-stage renal disease and is associated with adverse cardiovascular outcomes. Although the ankle–brachial index (ABI) is widely used to identify PAD, it may not fully reflect the complex vascular pathophysiology in patients undergoing peritoneal dialysis (PD). Antibodies against oxidized low-density lipoprotein (anti-oxLDL Ab) have been implicated in atherogenesis; however, their clinical relevance in PD populations remains unclear. *Materials and Methods*: In this cross-sectional investigation, 90 patients receiving maintenance PD were included. PAD was defined by an ABI below 0.90, and serum anti-oxLDL antibody concentrations were quantified using an enzyme-linked immunosorbent assay. *Results*: Patients with PAD were older (*p* = 0.006), had a higher prevalence of diabetes (*p* = 0.010), and exhibited higher levels of triglycerides (*p* = 0.008), fasting glucose (*p* < 0.001), and C-reactive protein (CRP, *p* < 0.001), but lower anti-oxLDL Ab levels (*p* = 0.008). Multivariable logistic regression demonstrated that reduced anti-oxLDL Ab levels (per 10 mU/mL increase, odds ratio [OR]: 0.803, 95% confidence interval [CI]: 0.648–0.995, *p* = 0.045) and increased CRP levels (per 0.1 mg/dL increase, OR: 1.662, 95% CI: 1.152–2.398, *p* = 0.007) were independently associated with PAD, with consistent results across penalized regression models. Log-transformed anti-oxLDL Ab levels were positively correlated with both left and right ABI values (*p* = 0.005 and *p* = 0.017, respectively). Decision curve analysis indicated that the anti-oxLDL Ab-based model provided greater net benefit compared with the treat-all and treat-none strategies across a range of threshold probabilities. *Conclusions*: Reduced serum anti-oxLDL Ab levels are independently associated with PAD in patients undergoing PD. Serum anti-oxLDL Ab levels are positively associated with ABI values. These findings suggest that impaired immunity against oxidized LDL may contribute to vascular disease in PD patients.

## 1. Introduction

Peripheral artery disease (PAD) is a progressive atherosclerotic disorder that commonly involves the abdominal aorta and lower-extremity arteries, leading to arterial stenosis or occlusion and consequent impairment of limb perfusion [1]. The prevalence of PAD is four- to six-fold higher in patients with end-stage renal disease (ESRD) compared with the general population and greatly contributes to morbidity, mortality, and healthcare costs [2,3]. PAD in ESRD increased the mortality risk by almost twofold and further increases in cases complicated by chronic limb-threatening ischemia [2]. These outcomes underscore the importance of early recognition and management of PAD in both nephrology and primary care settings [4]. The ankle–brachial index (ABI), which is the ratio of the ankle to brachial systolic blood pressure, is the most widely adopted non-invasive tool for PAD screening and is strongly associated with systemic atherosclerosis and mortality [5]. Additionally, a reduced ABI is correlated with systemic atherosclerosis and is a strong predictor of all-cause mortality in the general population [6]. In patients with ESRD, Miguel et al. revealed that a low ABI was an independent risk factor for mortality, underscoring its prognostic significance [7]. Nonetheless, relying on ABI alone may not fully capture the complex pathophysiological processes underlying vascular injury in patients undergoing dialysis, indicating the need for complementary biomarkers.

Oxidized low-density lipoprotein (oxLDL) plays an important role in the initiation and progression of atherosclerosis, while immune responses directed against oxLDL may reduce lesion formation and alter cardiovascular outcomes [8]. oxLDL is found in atherosclerotic plaques, where it activates lymphocytes, while antibodies targeting oxLDL are measurable in serum [9]. However, their clinical significance remains unclear. Higher circulating levels of oxLDL are reportedly associated with an increase in atherosclerotic burden and cardiovascular disease risk [10,11,12]. In contrast, other studies reported an inverse relationship, suggesting that these antibodies are protective [13,14,15].

Although PAD is highly prevalent in patients with ESRD, most previous studies have primarily focused on individuals undergoing maintenance hemodialysis, whereas evidence in patients receiving peritoneal dialysis (PD) remains limited. Given the distinct metabolic profiles, inflammatory status, and dialysis-related factors in PD populations, the relationship between antibodies against oxidized LDL (anti-oxLDL Ab) and PAD may differ from that observed in hemodialysis cohorts. However, data addressing this association in PD patients are scarce. To our knowledge, this relationship has not been specifically evaluated in a PD population. Therefore, we aimed to investigate the association between circulating anti-oxLDL Ab levels and ABI-defined PAD in patients undergoing PD.

## 2. Materials and Methods

### 2.1. Ethics and Study Participants

This cross-sectional study was conducted between 1 February and 31 May 2021, and included 90 patients on maintenance PD for >6 months due to ESRD at Hualien Tzu Chi Hospital, Taiwan. The study protocol was approved by the Institutional Review Board of Hualien Tzu Chi Hospital (IRB108-219-A). All participants provided written informed consent, and no financial incentives were offered. Individuals were excluded if they had ongoing infection, heart failure, acute coronary syndrome, prior stroke, malignancy, a history of limb amputation, or an ABI greater than 1.3. Clinical data on solute clearance, including weekly and peritoneal Kt/V and total and peritoneal creatinine clearance, were gathered from medical records. Diabetes mellitus was defined as a fasting plasma glucose level ≥ 126 mg/dL or the use of antidiabetic medications. Hypertension was defined as a systolic blood pressure ≥ 140 mmHg, a diastolic blood pressure ≥ 90 mmHg, or the use of antihypertensive agents within 2 weeks before study enrollment.

### 2.2. Anthropometric Analyses

Height and weight were measured thrice, with participants wearing light clothing and stockings. The standing height was measured from the soles of the feet to the vertex (Nagata H910 height-measuring device, Tainan, Taiwan). Body weight was measured with a digital scale (Hostart FM-200 electronic weighing scale, New Taipei City, Taiwan), and the mean of three readings was recorded and rounded to the nearest 0.5 cm or 0.5 kg. Body mass index, expressed as kg/m^2^, was calculated by normalizing body weight to the square of height.

### 2.3. Biochemical Tests

After fasting overnight for 8 h, 5 mL of venous blood was obtained before the daytime PD solution exchange; 0.5 mL was used for a complete blood count (XS-1000i hematology analyzer, Sysmex America, Mundelein, IL, USA). The residual sample was centrifuged at 3000× *g* for 10 min, and the resulting serum was stored at 4 °C for later analysis. Levels of serum cholesterol, triglycerides, fasting glucose, albumin, blood urea nitrogen, creatinine, calcium, phosphorus, and C-reactive protein (CRP) were determined using an autoanalyzer (Advia 1800 automated chemistry analyzer, Siemens Healthcare, Erlangen, Germany). Serum levels of intact parathyroid hormone (iPTH) and immunoglobulin G (IgG) antibodies against oxidized low-density lipoprotein (anti-oxLDL Ab) were measured using commercially available enzyme-linked immunosorbent assay kits (iPTH: NM59041, IBL International, Hamburg, Germany; anti-oxLDL Ab: BI-20032, Biomedica Immunoassays, Vienna, Austria) according to the manufacturers’ instructions. The anti-oxLDL Ab assay is a standardized indirect ELISA designed for the quantitative determination of circulating human IgG autoantibodies against oxLDL in serum. In this assay, microtiter wells are pre-coated with oxLDL antigen, allowing specific binding of anti-oxLDL Ab present in the sample, which are subsequently detected using a monoclonal anti-human IgG conjugated with horseradish peroxidase. The intra- and inter-assay coefficients of variation reported by the manufacturer were 3.6% and 2.8% for iPTH, and 4.8% and 6.0% for anti-oxLDL Ab, respectively, indicating acceptable assay precision.

### 2.4. ABI Measurements

ABI was evaluated with an automated oscillometric system (VaSera VS-1000; Fukuda Denshi, Tokyo, Japan). Systolic blood pressure was measured three times at both brachial arteries and both ankle arteries (dorsalis pedis and posterior tibial). Participants were in a supine position. To calculate the ABI, the highest systolic pressure in each ankle was divided by the highest brachial pressure. Each participant’s ABI was averaged from three consecutive measurements, and real-time electrocardiographic monitoring was performed for 15 min during the procedure. Device calibration was conducted according to the manufacturer’s instructions, and validated cuffs and airlines were used. PAD was diagnosed when the ABI was below 0.90 in at least one lower extremity, which is consistent with the established guidelines [16,17].

### 2.5. Statistical Analysis

A priori power analysis indicated that a minimum of 88 participants would provide 80% statistical power at a two-sided α level of 0.05 to detect a correlation coefficient of 0.30 between ABI and serum anti-oxLDL antibody levels. Continuous variables following a normal distribution are summarized as mean ± standard deviation and were compared using Student’s *t*-test. Variables with skewed distributions were reported as medians with interquartile ranges and analyzed using the Mann–Whitney U test. Categorical data are presented as counts with percentages and were evaluated using the chi-square test. Variables with skewed distributions, such as PD vintage and levels of triglycerides, glucose, iPTH, weekly Kt/V, CRP, and anti-oxLDL Ab, were log-transformed for correlation analysis. After adjusting for variables with significant differences between groups (age, diabetes, CRP, fasting glucose, triglycerides, and anti-oxLDL Ab), multivariable logistic regression was conducted to evaluate factors associated with PAD. These covariates were selected based on their clinical relevance and statistical significance in univariable analyses.

Given the relatively limited sample size and the number of covariates included in the models, penalized regression methods were applied to reduce the risk of overfitting and improve model stability. To further evaluate the robustness of the identified risk factors and address potential multicollinearity, penalized logistic regression analyses were performed. Three regularization methods were applied, including the Least Absolute Shrinkage and Selection Operator (LASSO), Ridge regression, and Elastic Net models. The same covariates included in the multivariable logistic regression analysis were entered into the penalized models (age, diabetes, CRP, fasting glucose, triglycerides, and anti-oxLDL Ab). To estimate the uncertainty of the regression coefficients and enhance model stability, a bootstrap resampling procedure with 1000 iterations was conducted to enhance the robustness of the estimates and provide more reliable confidence intervals. Adjusted odds ratios (ORs), 95% confidence intervals (CIs), and empirical *p*-values were derived from bootstrap distributions. Spearman’s correlation was used to evaluate relationships among the left and right ABIs, log-anti-oxLDL Ab levels, and clinical parameters.

Decision curve analysis (DCA) was applied to assess the potential clinical value of a prediction model incorporating serum anti-oxLDL antibody levels for PAD risk assessment. DCA estimates the net benefit of using a prediction model across a range of threshold probabilities by integrating the true-positive and false-positive rates while accounting for the relative harms of false-positive and false-negative decisions. The net benefit of the anti-oxLDL antibody-based model was compared with two default strategies: treating all patients and treating none. A higher net benefit of the model relative to these reference strategies across clinically relevant threshold probabilities was interpreted as evidence of potential clinical usefulness.

All analyses were performed using SPSS version 25.0 (IBM Corp., Armonk, NY, USA) and MedCalc version 22.019 (MedCalc Software, Ostend, Belgium), with two-tailed *p* < 0.05 considered statistically significant.

## 3. Results

### 3.1. Baseline Characteristics

Overall, 90 patients undergoing PD were enrolled and divided into the normal (*n* = 67) and low (*n* = 23; 25.6%) ABI groups. Table 1 presents the baseline characteristics of the participants. Participants with low ABIs were significantly older compared with those with normal ABIs (*p* = 0.006). Levels of triglycerides (*p* = 0.008), fasting glucose (*p* < 0.001), and CRP (*p* < 0.001) were higher in the low ABI group. In contrast, serum anti-oxLDL Ab levels were significantly lower in participants with low ABIs than those with normal ABIs (*p* = 0.008). The prevalence of diabetes was also higher among participants with low ABIs (*p* = 0.010). There were no significant differences between the groups in dialysis adequacy parameters or other biochemical indices.

### 3.2. Serum Anti-oxLDL Antibody Levels and PAD

Multivariable logistic regression analysis revealed that reduced anti-oxLDL Ab levels were independently associated with PAD (per 10 mU/mL increase, OR: 0.803, 95% CI: 0.648–0.995, *p* = 0.045). Additionally, increased CRP levels were associated with PAD (per 0.1 mg/dL increase, OR: 1.662, 95% CI: 1.152–2.398, *p* = 0.007). Meanwhile, diabetes, age, and fasting glucose and triglyceride levels were not significantly associated with PAD in the adjusted model (Table 2).

### 3.3. Penalized Logistic Regression Analysis

The results of the penalized logistic regression analyses are summarized in Table 3. Across all three models (LASSO, Ridge, and Elastic Net), serum anti-oxLDL Ab levels remained consistently and inversely associated with PAD. Although the effect size was modest, the adjusted ORs were consistently below unity and reached statistical significance in all models (bootstrap *p* < 0.05). Similarly, CRP demonstrated a robust positive association with PAD across all penalized regression models, with statistically significant adjusted ORs and narrow confidence intervals (all bootstrap *p* < 0.001), indicating that systemic inflammation remained a stable predictor after regularization. Triglyceride levels showed a small but positive association with PAD, reaching statistical significance in the Ridge and Elastic Net models, but not in the LASSO model. In contrast, diabetes mellitus status, age, and fasting glucose levels were not significantly associated with PAD after penalization in any of the three models. Overall, the associations for anti-oxLDL Ab and CRP were consistent across different penalization techniques.

### 3.4. Correlations Between Anti-oxLDL Antibody Levels and ABI

Spearman correlation analyses demonstrated that both left and right ABIs positively correlated with log-transformed anti-oxLDL Ab (log-anti-oxLDL Ab) levels (*r* = 0.293, *p* = 0.005, and *r* = 0.252, *p* = 0.017, respectively). Meanwhile, log-anti-oxLDL Ab levels were inversely correlated with age (*r* = −0.255, *p* = 0.015) and levels of total cholesterol (*r* = −0.237, *p* = 0.024), triglycerides (*r* = −0.362, *p* < 0.001), and log-CRP (*r* = −0.224, *p* = 0.034), whereas it was positively correlated with serum creatinine levels (*r* = 0.341, *p* = 0.001). Additionally, the left ABI values were negatively correlated with age (*r* = –0.341, *p* = 0.001), triglyceride levels (*r* = −0.346, *p* = 0.001), log-glucose levels (*r* = −0.374, *p* < 0.001), and log-CRP levels (*r* = −0.663, *p* < 0.001), while the right ABI values were negatively correlated with age (*r* = −0.223, *p* = 0.035), triglyceride levels (*r* = −0.377, *p* < 0.001), log-glucose levels (*r* = −0.412, *p* < 0.001), and log-CRP levels (*r* = −0.658, *p* < 0.001) (Table 4).

### 3.5. Decision Curve Analysis

**Figure 1:**
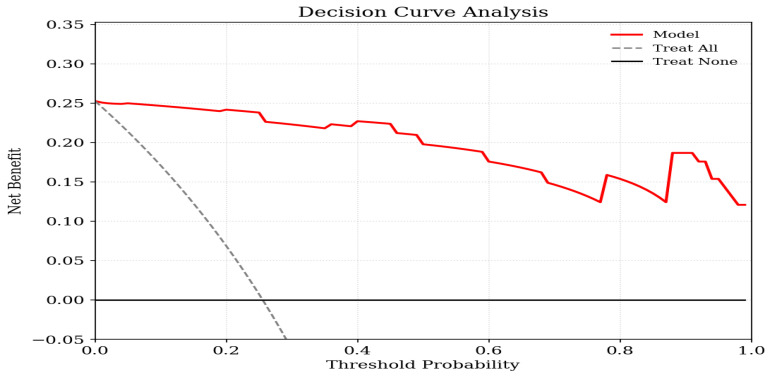
Decision curve analysis illustrating the clinical utility of the prediction model incorporating serum anti-oxLDL antibody levels. Net benefit is plotted on the vertical axis, while the horizontal axis represents the range of threshold probabilities. The solid red curve indicates the net benefit achieved by the anti-oxLDL antibody-based model. The reference strategies are shown by the gray dashed line, which assumes that all individuals are classified as having the outcome, and the black horizontal line, which assumes that no individuals have the outcome. Across a broad spectrum of threshold probabilities, the prediction model demonstrates superior net benefit compared with both reference approaches.

To evaluate the model’s clinical utility, we performed a DCA. As shown in Figure 1, the decision curve for the anti-oxLDL Ab-based model (red line) sits above the two reference lines (‘treat-all’ and ‘treat-none’) across a broad range of threshold probabilities. These findings indicate that the model including serum anti-oxLDL antibody levels yields greater net benefit than the treat-all or treat-none approaches across a clinically relevant range of threshold probabilities, supporting its potential value for PAD risk stratification.

## 4. Discussion

This study demonstrated that lower serum anti-oxLDL Ab levels were independently associated with PAD in patients undergoing PD. Patients undergoing PD with low ABIs not only had markedly reduced anti-oxLDL Ab levels but also had higher inflammatory activity, as reflected by elevated CRP levels, as well as a higher prevalence of diabetes. Multivariable regression analyses further supported these findings after adjustment for conventional risk factors. Correlation analyses further demonstrated that anti-oxLDL Ab levels were positively associated with ABI values and inversely associated with markers of dyslipidemia and inflammation. Although CRP showed a stronger association with PAD, anti-oxLDL Ab may provide additional insight by reflecting immune-mediated mechanisms distinct from systemic inflammation. These results support the hypothesis that impaired humoral immunity against oxLDL may play a role in the development of PAD among patients undergoing PD, highlighting its potential as a biomarker beyond traditional metabolic and inflammatory parameters. Importantly, given the cross-sectional design of this study, the observed relationships should be interpreted as associative rather than causal or predictive.

PAD is a manifestation of systemic atherosclerosis that significantly contributes to morbidity and mortality, especially in patients with ESRD [18]. The reversible and irreversible risk factors of PAD mostly overlap with those of ischemic heart disease [19]. Based on an ABI < 0.90, the prevalence of PAD ranges from 1.2% in a large managed care cohort of 6.67 million adults to approximately 29% in the PARTNERS study, which included older individuals and those with diabetes mellitus [20,21]. Similarly, our findings also confirmed that advanced age and diabetes were significantly associated with lower ABI values among patients undergoing PD. Furthermore, triglycerides are well-established markers of atherogenic dyslipidemia [22], and elevated levels are associated with progression of atherosclerosis [23]. In this study, we determined that elevated triglyceride levels were inversely correlated with ABI values. Furthermore, inflammation plays a central role in the pathogenesis of cardiovascular disease [24]. CRP consistently predicts future cardiovascular events in large prospective cohorts [25,26]. Using data from the National Health and Nutrition Examination Survey, Shankar et al. reported that elevated CRP levels are independently associated with PAD in the general US population [27]. Our findings confirm these reports, as our results revealed that CRP levels were strongly and inversely associated with ABI values in our cohort.

Beyond these conventional risk factors, our study underscores the potential role of immune responses to oxidized lipoproteins in the development of PAD in patients undergoing PD. Notably, serum anti-oxLDL Ab levels were significantly lower in patients undergoing PD with PAD, and these reduced levels remained independently associated with PAD even after adjusting for traditional risk factors. To further address potential overfitting and multicollinearity due to the limited sample size, we applied penalized logistic regression models as sensitivity analyses. Importantly, the inverse association between anti-oxLDL Ab levels and PAD, as well as the positive association for CRP, remained consistent across LASSO, Ridge, and Elastic Net models. The consistency of findings across different penalized regression models supports the robustness of the observed associations. These approaches are particularly relevant in studies with limited sample sizes, where conventional regression models may be prone to overfitting. Moreover, anti-oxLDL Ab levels were positively correlated with ABI values and inversely correlated with triglycerides and CRP levels, indicating that impaired humoral immunity against oxLDL may reflect a state of heightened dyslipidemia and inflammation. These findings suggest that reduced antibody-mediated clearance of oxLDL may accelerate atherogenesis in patients on dialysis, extending the understanding of vascular risk beyond established metabolic and inflammatory markers. Although the odds ratio for anti-oxLDL Ab was close to unity when expressed per 1 mU/mL increase, this reflects the small unit scale of measurement. Given the wide range of anti-oxLDL Ab levels observed in this cohort, even modest variations across the physiological range may be associated with clinically meaningful differences. For example, a difference of 100 mU/mL in anti-oxLDL Ab levels may correspond to a more substantial change in estimated risk.

The mechanisms underlying the association between decreased anti-oxLDL Ab levels and PAD in patients on dialysis remain unclear. Antibodies against oxLDL may facilitate oxLDL clearance [10] and attenuate foam cell formation [28], thereby being protective against atherosclerosis. Regarding ESRD, chronic exposure to oxidative stress and systemic inflammation impairs humoral immunity, leading to reduced antibody production or elevated antibody consumption due to persistent antigenic stimulation [29,30]. The imbalance may diminish the body’s capacity to neutralize oxLDL, leading to increased vascular injury and progression of atherosclerotic lesions [31]. The inverse correlations we observed among anti-oxLDL Ab, triglyceride, and CRP levels indicate that dyslipidemia and inflammation may act synergistically with impaired immunity to accelerate PAD development and progression in patients undergoing PD. This is consistent with observations in autoimmune inflammatory diseases, wherein a proatherogenic dyslipidemic state that is characterized by dysfunctional high-density lipoproteins and elevated oxLDL levels further increases the cardiovascular risk [32]. As patients on PD experience persistent low-grade inflammation, oxidative stress, and immune dysregulation [33], these states may further enhance the detrimental vascular effects of reduced anti-oxLDL Ab levels in this population.

Clinically, measuring anti-oxLDL Ab levels is relatively simple and cost-effective compared to imaging modalities. While ABI remains the standard tool for PAD detection, its accuracy in patients undergoing dialysis may be limited by vascular calcification and arterial stiffness [34,35]. In this clinical setting, anti-oxLDL Ab may serve as a complementary biomarker alongside established markers such as CRP, reflecting immune-mediated mechanisms that are not fully captured by systemic inflammatory markers. Rather than serving as a standalone diagnostic or predictive tool, anti-oxLDL Ab may provide additional information for risk stratification, particularly in identifying patients with higher vascular risk within the PD population. DCA further suggested that incorporating anti-oxLDL Ab levels into the model may provide additional information for risk assessment compared with default strategies across a range of threshold probabilities. However, these findings remain exploratory, and the clinical applicability of anti-oxLDL Ab measurement requires further validation in larger, prospective studies before integration into routine clinical practice can be considered.

This study has several limitations. First, the cross-sectional design prevents conclusions regarding causal relationships between anti-oxLDL Ab levels and PAD. Second, this study was performed in a single center and had a relatively small sample size, which may limit the generalizability of our results. In addition, the relatively small sample size and inclusion of multiple covariates may increase the risk of overfitting. Although we applied penalized regression models and bootstrap resampling to mitigate this issue, the findings should still be interpreted with caution and require validation in larger cohorts. Third, PAD was defined solely based on ABI measurements, without confirmatory imaging such as duplex ultrasound or computed tomography angiography. In patients undergoing dialysis, ABI may be affected by medial arterial calcification, potentially leading to falsely elevated values and misclassification of PAD status. Therefore, the use of ABI alone may have resulted in an underestimation of PAD prevalence in this population. Additionally, we did not assess the longitudinal changes in anti-oxLDL Ab levels or their correlation with cardiovascular outcomes such as myocardial infarction, limb amputation, or mortality. Future research involving larger multicenter cohorts and prospective follow-up is warranted to validate these findings and to clarify the prognostic significance of anti-oxLDL Ab. Furthermore, mechanistic investigations into the interplay among lipid metabolism, immune dysregulation, and vascular injury in patients on dialysis are warranted to better understand the pathophysiological role of these antibodies and to determine their potential utility as therapeutic targets.

## 5. Conclusions

In conclusion, reduced serum anti-oxLDL antibody levels were independently associated with PAD in patients undergoing PD, alongside established risk factors such as age, diabetes, dyslipidemia, and inflammation; however, these findings should be interpreted as associative rather than causal. Although CRP showed the strongest association with PAD, anti-oxLDL Ab levels provided complementary information reflecting immune-mediated mechanisms distinct from systemic inflammation. Integrating ABI with inflammatory and immune-related biomarkers may provide additional information for risk stratification in this high-risk population.

## Figures and Tables

**Table 1:** Clinical variables of the 90 peritoneal dialysis patients in the normal or low ankle brachial index group.

Characteristic	All Participants (*n* = 90)	Normal ABI Group (*n* = 67)	Low ABI Group (*n* = 23)	*p* Value
Age (years)	57.89 ± 14.61	55.45 ± 14.95	65.00 ± 11.06	0.006 *
PD duration (months)	48.57 (21.09–81.15)	37.80 (20.16–70.08)	62.88 (25.56–96.12)	0.085
Height (cm)	160.32 ± 8.85	160.48 ± 8.64	159.87 ± 9.60	0.778
Body weight (kg)	64.88 ± 14.22	64.00 ± 13.93	67.44 ± 15.05	0.318
Body mass index (kg/m^2^)	25.11 ± 4.43	24.74 ± 4.50	26.17 ± 4.14	0.184
Left ABI	1.02 ± 0.15	1.09 ± 0.08	0.83 ± 0.13	<0.001 *
Right ABI	1.04 ± 0.16	1.11 ± 0.09	0.83 ± 0.14	<0.001 *
SBP (mmHg)	144.42 ± 19.08	145.30 ± 16.83	141.87 ± 24.78	0.460
DBP (mmHg)	84.29 ± 9.80	85.22 ± 9.32	81.57 ± 10.86	0.123
Hemoglobin (g/dL)	9.66 ± 1.37	9.50 ± 1.43	10.11 ± 1.11	0.066
Albumin (g/dL)	3.54 ± 0.34	3.51 ± 0.37	3.62 ± 0.26	0.199
Total cholesterol (mg/dL)	155.02 ± 47.88	157.54 ± 43.86	147.70 ± 58.53	0.398
Triglycerides (mg/dL)	120.50 (81.25–196.00)	107.00 (77.00–164.00)	186.00 (112.00–331.00)	0.008 *
Fasting glucose (mg/dL)	102.00 (91.00–124.00)	98.00 (89.00–110.00)	130.00 (100.00–165.00)	<0.001 *
Blood urea nitrogen (mg/dL)	60.67 ± 17.69	61.49 ± 18.56	58.26 ± 14.99	0.453
Creatinine (mg/dL)	10.40 ± 3.31	10.60 ± 3.51	9.84 ± 2.66	0.348
Total calcium (mg/dL)	9.63 ± 0.61	9.58 ± 0.64	9.75 ± 0.50	0.258
Phosphorus (mg/dL)	5.24 ± 1.31	5.30 ± 1.36	5.07 ± 1.17	0.468
iPTH (pg/mL)	192.45 (80.53–452.78)	194.90 (80.80–447.00)	190.00 (74.20–470.10)	0.850
C-reactive protein (mg/dL)	0.18 (0.11–1.22)	0.14 (0.10–0.24)	2.52 (1.50–4.47)	<0.001 *
Anti-oxLDL Ab (mU/mL)	269.10 (118.60–496.13)	307.30 (136.90–601.80)	143.20 (91.32–352.00)	0.008 *
Weekly Kt/V	1.97 (1.71–2.17)	1.98 (1.72–2.18)	1.85 (1.58–2.06)	0.144
Peritoneal Kt/V	1.76 ± 0.39	1.75 ± 0.40	1.77 ± 0.38	0.875
Total Clcr (L/week)	58.05 ± 15.83	59.78 ± 17.36	53.01 ± 8.59	0.077
Peritoneal Clcr (L/week)	47.14 ± 12.27	47.01 ± 12.88	47.51 ± 10.53	0.868
Female, *n* (%)	51 (56.7)	39 (58.2)	12 (52.2)	0.614
Diabetes, *n* (%)	38 (42.2)	23 (34.3)	15 (65.2)	0.010 *
Hypertension, *n* (%)	71 (78.9)	53 (79.1)	18 (78.3)	0.932
CAPD model, *n* (%)	30 (33.3)	23 (34.3)	7 (30.4)	0.733
Smoking, *n* (%)	8 (8.9)	5 (7.5)	3 (13.0)	0.417
ARB use, *n* (%)	53 (58.9)	42 (62.7)	11 (47.8)	0.211
β-blocker use, *n* (%)	35 (38.9)	28 (41.8)	7 (30.4)	0.335
CCB use, *n* (%)	48 (53.3)	38 (56.7)	10 (43.5)	0.272
Statin use, *n* (%)	31 (34.4)	23 (34.3)	8 (34.8)	0.968
Fibrate use, *n* (%)	15 (16.7)	10 (14.9)	5 (21.7)	0.449

Continuous data are reported as mean ± standard deviation for variables with a normal distribution and were compared using Student’s *t*-test. Variables showing non-normal distributions were presented as medians with interquartile ranges and analyzed using the Mann–Whitney U test. Categorical data are expressed as counts with percentages and were compared using the chi-square test. Abbreviations: PD, peritoneal dialysis; ABI, ankle–brachial index; SBP, systolic blood pressure; DBP, diastolic blood pressure; iPTH, intact parathyroid hormone; anti-oxLDL Ab, antibodies against oxidized low-density lipoprotein; Kt/V, fractional clearance index for urea; Clcr, creatinine clearance; CAPD, continuous ambulatory peritoneal dialysis; ARB, angiotensin receptor blocker; CCB, calcium channel blocker. * *p* < 0.05 was considered statistically significant.

**Table 2:** Factors associated with peripheral arterial disease identified by multivariable logistic regression in 90 patients undergoing peritoneal dialysis.

Variables	Odds Ratio	95% CI	*p* Value
Anti-oxLDL antibodies (10 mU/mL)	0.803	0.648–0.995	0.045 *
C-reactive protein (0.1 mg/dL)	1.662	1.152–2.398	0.007 *
Diabetes mellitus (present)	1.584	0.047–52.595	0.797
Age (1 year)	0.998	0.898–1.110	0.978
Fasting glucose (1 mg/dL)	1.016	0.970–1.064	0.499
Triglyceride (1 mg/dL)	1.004	0.996–1.012	0.349

Multivariable logistic regression was applied, with diabetes status, age, C-reactive protein (per 0.1 mg/dL), fasting glucose, triglyceride levels, and serum anti-oxLDL antibodies (per 10 mU/mL) included as covariates. CI indicates confidence interval; anti-oxLDL, antibodies against oxidized low-density lipoprotein. * *p* < 0.05 was considered statistically significant.

**Table 3:** Results of penalized logistic regression analyses for factors associated with peripheral arterial disease, expressed as adjusted odds ratios with 95% confidence intervals.

Factors	LASSO OR (95% CI)	LASSO *p* Value	Ridge OR (95% CI)	Ridge *p* Value	Elastic Net OR (95% CI)	Elastic Net *p* Value
Anti-oxLDL Abs (1 mU/mL)	0.994 (0.990, 1.000)	0.048 *	0.997 (0.995, 0.999)	<0.001 *	0.996 (0.994, 0.999)	0.005 *
C-reactive protein (0.1 mg/dL)	1.274 (1.204, 1.469)	<0.001 *	1.188 (1.135, 1.278)	<0.001*	1.214 (1.157, 1.318)	<0.001 *
Diabetes mellitus (present)	2.466 (1.000, 23.948)	0.343	2.452 (0.627, 11.270)	0.188	2.507 (0.829, 13.575)	0.233
Age (1 year)	1.009 (0.971, 1.091)	0.945	1.020 (0.970, 1.081)	0.487	1.016 (0.971, 1.084)	0.652
Fasting glucose (1 mg/dL)	1.010 (0.985, 1.039)	0.595	1.010 (0.989, 1.035)	0.287	1.010 (0.989, 1.036)	0.430
Triglyceride (1 mg/dL)	1.003 (1.000, 1.013)	0.060	1.004 (1.001, 1.011)	0.018 *	1.004 (1.000, 1.012)	0.025 *

To validate the stability of the identified risk factors and mitigate potential overfitting due to the sample size, we performed penalized logistic regression analyses using LASSO, Ridge, and Elastic Net regularization. To estimate the uncertainty of the model coefficients, we utilized a bootstrap resampling procedure with 1000 iterations. The 95% CIs and empirical *p*-values for the odds ratios were derived from bootstrap distributions. Abbreviations: LASSO, least absolute shrinkage and selection operator; OR, odds ratio; CI, confidence interval. * *p* < 0.05 was considered statistically significant (2-tailed).

**Table 4:** Associations of left and right ankle–brachial index values and log-transformed serum anti-oxLDL antibody levels with clinical parameters in 90 patients undergoing peritoneal dialysis.

Variables	ABI (Left)		ABI (Right)		Log-Anti-oxLDL Ab (mU/mL)	
	Spearman’s Rho	*p* Value	Spearman’s Rho	*p* Value	Spearman’s Rho	*p* Value
Age (years)	−0.341	0.001 *	−0.223	0.035 *	−0.255	0.015 *
Body mass index (kg/m^2^)	−0.188	0.076	−0.097	0.364	0.009	0.935
Log-PD vintage (months)	0.030	0.776	−0.077	0.474	0.080	0.453
Left ABI	—	—	0.753	<0.001 *	0.293	0.005 *
Right ABI	0.753	<0.001 *	—	—	0.252	0.017 *
Log-anti-oxLDL Ab (mU/mL)	0.293	0.005 *	0.252	0.017 *	**—**	**—**
SBP (mmHg)	0.076	0.478	0.138	0.195	−0.080	0.455
DBP (mmHg)	0.192	0.070	0.191	0.071	0.075	0.480
Hemoglobin (g/dL)	−0.117	0.270	−0.179	0.091	−0.039	0.712
Albumin (g/dL)	−0.096	0.369	−0.089	0.405	−0.019	0.861
Total cholesterol (mg/dL)	−0.001	0.996	0.068	0.525	−0.237	0.024 *
Triglyceride (mg/dL)	−0.346	0.001 *	−0.377	<0.001 *	−0.362	<0.001 *
Log-Glucose (mg/dL)	−0.374	<0.001 *	−0.412	<0.001 *	−0.154	0.147
BUN (mg/dL)	0.011	0.920	0.109	0.308	0.083	0.436
Creatinine (mg/dL)	0.148	0.160	0.159	0.133	0.341	0.001 *
Total calcium (mg/dL)	−0.085	0.424	−0.149	0.160	0.110	0.303
Phosphorus (mg/dL)	0.032	0.761	0.054	0.615	0.186	0.079
Log-iPTH (pg/mL)	0.086	0.422	0.084	0.428	0.114	0.285
Log-CRP (mg/L)	−0.663	<0.001 *	−0.658	<0.001*	−0.224	0.034 *
Log-Weekly Kt/V	0.124	0.246	0.095	0.375	−0.176	0.097
Peritoneal Kt/V	0.017	0.876	−0.079	0.461	−0.017	0.873
Total Clcr (L/week)	0.117	0.164	0.164	0.123	−0.020	0.849
Peritoneal Clcr (L/week)	−0.007	0.949	0.001	0.994	0.162	0.127

Data on PD vintage, triglycerides, glucose, iPTH, Weekly Kt/V, and anti-oxLDL Ab levels showed skewed distributions and were therefore log-transformed before analysis. Abbreviations: ABI, ankle brachial index; Anti-oxLDL Ab, anti-oxidized low-density lipoprotein antibodies; PD, peritoneal dialysis; SBP, systolic blood pressure; DBP, diastolic blood pressure; BUN, blood urea nitrogen; iPTH, Intact parathyroid hormone; Kt/V, fractional clearance index for urea; Clcr, clearance of creatinine. * *p* < 0.05 was considered statistically significant (2-tailed).

## Data Availability

The data presented in this study are available on request from the corresponding author, as public access is restricted to protect patient privacy and comply with ethical requirements.

## Abbreviations

The following abbreviations are used in this manuscript:

ABI	Ankle−brachial index
Anti-oxLDL Ab	Antibodies against oxLDL
BMI	Body mass index
CI	Confidence interval
CRP	C-reactive protein
DCA	Decision curve analysis
ESRD	End-stage renal disease
iPTH	Intact parathyroid hormone
LASSO	Least absolute shrinkage and selection operator
OR	Odds ratio
oxLDL	Oxidized low-density lipoprotein
PAD	Peripheral artery disease
PD	Peritoneal dialysis

## References

[1] Huish S., Nawaz S., Bellasi A., Diaz-Tocados J.M., Haarhaus M., Sinha S. (2025). Clinical management of peripheral arterial disease in chronic kidney disease-a comprehensive review from the European Renal Association CKD-MBD Working Group. Clin. Kidney J..

[2] De Stefano F., Rios L.H.P., Fiani B., Fareed J., Tafur A. (2021). National trends for peripheral artery disease and end stage renal disease from the National Inpatient Sample Database. Clin. Appl. Thromb. Hemost..

[3] Ho C.L.B., Chih H.J., Garimella P.S., Matsushita K., Jansen S., Reid C.M. (2021). Prevalence and risk factors of peripheral artery disease in a population with chronic kidney disease in Australia: A systematic review and meta-analysis. Nephrology.

[4] DeLoach S.S., Mohler E.R. (2007). Peripheral arterial disease: A guide for nephrologists. Clin. J. Am. Soc. Nephrol..

[5] Aboyans V., Criqui M.H., Abraham P., Allison M.A., Creager M.A., Diehm C., Fowkes F.G., Hiatt W.R., Jönsson B., Lacroix P. (2012). Measurement and interpretation of the ankle-brachial index: A scientific statement from the American Heart Association. Circulation.

[6] Newman A.B., Shemanski L., Manolio T.A., Cushman M., Mittelmark M., Polak J.F., Powe N.R., Siscovick D. (1999). Ankle-arm index as a predictor of cardiovascular disease and mortality in the Cardiovascular Health Study. The Cardiovascular Health Study Group. Arterioscler. Thromb. Vasc. Biol..

[7] Miguel J.B., Matos J.P.S., Lugon J.R. (2017). Ankle-brachial index as a predictor of mortality in hemodialysis: A 5-year cohort study. Arq. Bras. Cardiol..

[8] Matsuura E., Hughes G.R., Khamashta M.A. (2008). Oxidation of LDL and its clinical implication. Autoimmun. Rev..

[9] Virella G., Virella I., Leman R.B., Pryor M.B., Lopes-Virella M.F. (1993). Anti-oxidized low-density lipoprotein antibodies in patients with coronary heart disease and normal healthy volunteers. Int. J. Clin. Lab. Res..

[10] Shoji T., Fukumoto M., Kimoto E., Shinohara K., Emoto M., Tahara H., Koyama H., Ishimura E., Nakatani T., Miki T. (2002). Antibody to oxidized low-density lipoprotein and cardiovascular mortality in end-stage renal disease. Kidney Int..

[11] Shoenfeld Y., Wu R., Dearing L.D., Matsuura E. (2004). Are anti-oxidized low-density lipoprotein antibodies pathogenic or protective?. Circulation.

[12] Bergmark C., Wu R., de Faire U., Lefvert A.K., Swedenborg J. (1995). Patients with early-onset peripheral vascular disease have increased levels of autoantibodies against oxidized LDL. Arterioscler. Thromb. Vasc. Biol..

[13] van den Berg V.J., Vroegindewey M.M., Kardys I., Boersma E., Haskard D., Hartley A., Khamis R. (2019). Anti-oxidized LDL antibodies and coronary artery disease: A systematic review. Antioxidants.

[14] Fukumoto M., Shoji T., Emoto M., Kawagishi T., Okuno Y., Nishizawa Y. (2000). Antibodies against oxidized LDL and carotid artery intima-media thickness in a healthy population. Arterioscler. Thromb. Vasc. Biol..

[15] Shoji T., Nishizawa Y., Fukumoto M., Shimamura K., Kimura J., Kanda H., Emoto M., Kawagishi T., Morii H. (2000). Inverse relationship between circulating oxidized low density lipoprotein (oxLDL) and anti-oxLDL antibody levels in healthy subjects. Atherosclerosis.

[16] Hsu B.G., Wang C.H., Lai Y.H., Kuo C.H., Lin Y.L. (2024). Association of endothelial dysfunction and peripheral arterial disease with sarcopenia in chronic kidney disease. J. Cachexia Sarcopenia Muscle.

[17] Chern Y.B., Lee P.S., Wang J.H., Tsai J.P., Hsu B.G. (2025). Increased serum sclerostin level is a risk factor for peripheral artery disease in patients with hypertension. Medicina.

[18] O’Hare A., Johansen K. (2001). Lower-extremity peripheral arterial disease among patients with end-stage renal disease. J. Am. Soc. Nephrol..

[19] Bartholomew J.R., Olin J.W. (2006). Pathophysiology of peripheral arterial disease and risk factors for its development. Clevel. Clin. J. Med..

[20] Margolis J., Barron J.J., Grochulski W.D. (2005). Health care resources and costs for treating peripheral artery disease in a managed care population: Results from analysis of administrative claims data. J. Manag. Care Pharm..

[21] Hirsch A.T., Criqui M.H., Treat-Jacobson D., Regensteiner J.G., Creager M.A., Olin J.W., Krook S.H., Hunninghake D.B., Comerota A.J., Walsh M.E. (2001). Peripheral arterial disease detection, awareness, and treatment in primary care. JAMA.

[22] Arca M., Montali A., Valiante S., Campagna F., Pigna G., Paoletti V., Antonini R., Barillà F., Tanzilli G., Vestri A. (2007). Usefulness of atherogenic dyslipidemia for predicting cardiovascular risk in patients with angiographically defined coronary artery disease. Am. J. Cardiol..

[23] Pop-Busui R., Boulton A.J., Feldman E.L., Bril V., Freeman R., Malik R.A., Sosenko J.M., Ziegler D. (2017). Diabetic neuropathy: A position statement by the American Diabetes Association. Diabetes Care.

[24] Lucas A.R., Korol R., Pepine C.J. (2006). Inflammation in atherosclerosis: Some thoughts about acute coronary syndromes. Circulation.

[25] Danesh J., Wheeler J.G., Hirschfield G.M., Eda S., Eiriksdottir G., Rumley A., Lowe G.D., Pepys M.B., Gudnason V. (2004). C-reactive protein and other circulating markers of inflammation in the prediction of coronary heart disease. N. Engl. J. Med..

[26] Koenig W., Löwel H., Baumert J., Meisinger C. (2004). C-reactive protein modulates risk prediction based on the Framingham Score: Implications for future risk assessment: Results from a large cohort study in southern Germany. Circulation.

[27] Shankar A., Li J., Nieto F.J., Klein B.E., Klein R. (2007). Association between C-reactive protein level and peripheral arterial disease among US adults without cardiovascular disease, diabetes, or hypertension. Am. Heart J..

[28] Hörkkö S., Bird D.A., Miller E., Itabe H., Leitinger N., Subbanagounder G., Berliner J.A., Friedman P., Dennis E.A., Curtiss L.K. (1999). Monoclonal autoantibodies specific for oxidized phospholipids or oxidized phospholipid-protein adducts inhibit macrophage uptake of oxidized low-density lipoproteins. J. Clin. Investig..

[29] Kato S., Chmielewski M., Honda H., Pecoits-Filho R., Matsuo S., Yuzawa Y., Tranaeus A., Stenvinkel P., Lindholm B. (2008). Aspects of immune dysfunction in end-stage renal disease. Clin. J. Am. Soc. Nephrol..

[30] Chi M., Tian Z., Ma K., Li Y., Wang L., Nasser M.I., Liu C. (2022). The diseased kidney: Aging and senescent immunology. Immun. Ageing.

[31] Poznyak A.V., Nikiforov N.G., Markin A.M., Kashirskikh D.A., Myasoedova V.A., Gerasimova E.V., Orekhov A.N. (2021). Overview of oxLDL and its impact on cardiovascular health: Focus on atherosclerosis. Front. Pharmacol..

[32] Wilkinson M.J., Shapiro M.D. (2024). Immune-mediated inflammatory diseases, dyslipidemia, and cardiovascular risk: A complex interplay. Arterioscler. Thromb. Vasc. Biol..

[33] Innico G., Gobbi L., Bertoldi G., Rigato M., Basso A., Bonfante L., Calò L.A. (2021). Oxidative stress, inflammation, and peritoneal dialysis: A molecular biology approach. Artif. Organs.

[34] AbuRahma A.F., Adams E., AbuRahma J., Mata L.A., Dean L.S., Caron C., Sloan J. (2020). Critical analysis and limitations of resting ankle-brachial index in the diagnosis of symptomatic peripheral arterial disease patients and the role of diabetes mellitus and chronic kidney disease. J. Vasc. Surg..

[35] Chen J., He H., Starcke C.C., Guo Y., Geng S., Chen C.S., Mahone E.B., Batuman V., Hamm L.L., He J. (2021). Accuracy of ankle-brachial index, toe-brachial index, and risk classification score in discriminating peripheral artery disease in patients with chronic kidney disease. Am. J. Cardiol..

